# Changing priorities about protective shelters: a review of a key method to investigate possible pain in crustaceans

**DOI:** 10.1098/rsbl.2025.0342

**Published:** 2025-09-17

**Authors:** Robert William Elwood

**Affiliations:** ^1^ School of Biological Sciences, Queen’s University Belfast, Belfast BT9 5DL, UK

**Keywords:** priorities, shelters, pain, decapods, trade-off

## Abstract

Testing if non-human taxa experience pain is difficult because we need to exclude the possibility that responses are nociceptive reflexes. One approach is to identify an essential, high priority, resource and then ask if the animal will abandon and subsequently avoid that resource if it is paired with a noxious stimulus. This approach has been used with crustaceans that hide in dark shelters and electric shocks have been used as noxious stimuli. A range of species show escape responses and avoid shelters if the shock is presented within, and these responses increase with increasing voltage or repetition of shocks. Crustaceans also switch to using alternative shelters and appear to dramatically alter their behavioural priorities. Animals shocked outside of a shelter, however, subsequently increase their use of shelters and benefit from reduced predation. These changes in priorities cannot be due only to nociceptive reflexes because they persist long after the cessation of the stimulus. Increasing the apparent costs of leaving a shelter decreases the probability of leaving, indicating that, by taking into account costs, they are responding via behavioural decisions and not reflexes. This provides a method to determine how much the animal will pay to avoid the shocks and similar techniques should provide powerful ways to examine potential pain in different taxa.

## 1. Introduction

There has been considerable debate about which taxa might be sentient, especially with respect to pain experience [1–10]. However, absolute certainty about pain is impossible because we cannot access the private minds of non-human animals. Nevertheless, lack of absolute proof should not dissuade us from seeking evidence both for and against the idea of pain. In this task we need to consider predictions about how pain might alter behaviour, and test if those predictions are upheld or not [1,5]. The aim is to enable a consensus about the possibility of pain experience in each taxon. Only if we can make informed judgements can we assess how to minimize suffering. That is, if we suspect pain might occur, then we should use the precautionary principle and offer the animals the benefit of doubt [4,11].

Most metazoans have specialized receptors for detecting noxious stimuli that cause potential or actual tissue damage. These nociceptors initiate a reflex response that removes all or part of the animal from the stimulus, thus providing immediate cessation of tissue damage. The reflex response does not require any centrally processed decision-making, and feelings by the animal are unlikely [4]. However, humans have additional connections from the nociceptors to the brain where specialized circuits generate the highly unpleasant emotional experience that is the feeling of pain [6]. For pain to have evolved we expect the costs of developing the neural circuits to be offset by some benefits. While a nociceptive reflex might offer immediate escape from the noxious stimulus the lack of feeling or awareness means little or no lasting effects. By contrast, pain is expected to change behaviour to reduce future tissue damage, and elicit activities that enhance healing of a wound, which should provide a fitness advantage over reflex responses [5]. With pain, we expect to see avoidance of activities or locations that are associated with the noxious stimulus. That is, pain is expected to rearrange the motivational priorities of an animal. By contrast, a nociceptive reflex could occur without any feeling, experience or awareness of the situation, and subsequent changes in priorities are unlikely.

This key distinction between pain and nociceptive reflexes provides targets for experiments on the possibility of pain [12]. One influential approach is to identify an activity that is normally a major priority for the taxon being assessed, and to see if noxious stimuli cause the animals to reduce or give up that priority to avoid the noxious stimuli. Feeding, drinking and mating are high priority activities, but in this review I focus on predator avoidance by shelter use. Leaving a shelter and, thus, not avoiding predators, even for short durations, could have major negative fitness consequences, more so than might occur by not feeding or drinking for similar short durations. Many species use shelters or burrows to hide from predators, and a noxious stimulus may be experimentally delivered within the shelter to assess if shelter use is negatively impacted. If the animal leaves the shelter it would be giving up a valuable resource and taking risks to avoid the noxious stimulus. We might also increase stimuli associated with the value of the resource and predation risk to see if animals will still leave a shelter even though predator cues indicate a high risk. If these features alter the response of the animal then we have a trade-off between resource value and shock avoidance and this indicates a behavioural decision rather than a reflex [4,5,9,12]. If we see animals taking risks to avoid the noxious stimulus, especially if that continues after the cessation of the stimulus, then we can infer that avoiding the stimulus has a high priority. The present paper reviews experiments that examine changes in shelter use in various crustaceans following electric shock. Electric shocks have been used as noxious stimuli because of the ease of applying the stimulus when the animal is behaving normally, and the stimulus can be repeated with a set duration, frequency and voltage. That is, the stimulus can be replicated with precision. Here, studies on various species, with different original aims but with similarities in methods, are compared to gain insights into the question of pain beyond that gained from each separate study.

## 2. Shocks within immobile shelters

Shore crabs, *Carcinus maenas*, hide from visual predators by inhabiting dark crevices under rocks during daytime low tides. Such crevices have been replicated in several experiments by using a brightly lit arena and a dark, low shelter in which the crab can hide [13–16]. The use of dark shelters by shore crabs has been shown to be a high priority because, when placed in the arena, all crabs quickly leave the light and go to the dark shelter. If the crab is removed from the shelter and returned to the light it typically enters the shelter again so repeated trials are possible. Without further intervention crabs remain in the shelter for the whole trial (2 min in these studies), again indicating the high priority given to hiding in the dark. However, electric shocks may be applied to some crabs within the shelter to determine if that induces emergence from the dark into the light, and, in subsequent trials, if prior shocks reduce the number of crabs that enter the shelter or cause hesitation before shelter entry, or induce crabs to switch to an alternative shelter. If the data show such effects, especially long-term effects, then that would be consistent with the idea of pain, however, if they fail to show the long-term effects then that would be consistent with the idea of reflex responses and not pain [12].

As an example of this, Barr & Elwood [13] placed crabs in an arena in 20 trials and a single 8 V shock (180 Hz, 200 ms) was delivered to the joints of the 5^th^ pereiopods/walking legs of some crabs when they entered the shelter. Shelter use was compared with crabs that were not shocked but the small decline in the probability of shelter entry by those that were shocked was not significant. However, the latency to enter the shelter increased for the shocked crabs whereas it decreased over trials for the non-shocked crabs. Further, only animals that had received a shock emerged from the shelter. That is, these mild shocks caused some crabs to give up their normal high priority use of a shelter. In this experiment we cannot exclude the possibility that abandoning the shelter is a reflex. However, shocks also induced hesitation about shelter entry and this cannot be a reflex response.

A second study also gave a single shock but of either 6 or 12 V (180 Hz, 200 ms), or no shock, and also varied the level of light in the arena (180 or 2060 lx) [14]. There was a significant effect of shock on shelter entry, particularly in later trials, with fewer going in if they had experienced the 12 V shock. There were more shelter entries from the bright light than from the dull light, particularly in later trials. The latency to enter the shelter was greater for crabs receiving shocks and this was more marked in the 12 V group, and shocked crabs were seen to back away from the shelter and push against the walls of the tank before moving to the shelter, again especially with the 12 V group. Six crabs that received the 12 V shock emerged from the shelter on one or more occasions, but this was not significantly affected by light levels. Considering all measures, the priority for shelter use was markedly reduced when voltage was high but increased when the light was bright. When shock was high, crabs seemed to attempt to use a different way of seeking shelter and only went to the shelter when that failed [14].

A further two studies investigated avoidance learning in shore crabs, using an arena that had two shelters. Entry of one shelter resulted in a 10 V shock (180 Hz, 200 ms) whereas entry to the other resulted in no shock over 10 trials [15,16]. A key difference between these studies and those noted above was that shocks were delivered 5 s after entry and were repeated every 5 s if the crab remained in the shelter. In the first study, 50–60% of crabs emerged from the shock shelter in trials 1−5 and over 80% in trials 6−10, which was far higher than seen in studies that used a single shock within each trial [15]. No crab emerged without being shocked, which indicates the normal priority of remaining in the dark shelter. Crabs showed a strong preference for returning to the same shelter in the second trial and this was not affected by whether they had been shocked in the first trial. On the next trial, however, those that had received shocks were more likely to switch their choice to the alternative shelter. Those that switched following shocks demonstrated avoidance learning but, to do so, they had to overcome an initial preference. The latency to emerge from a shelter did not change over the trials.

The second learning experiment had a barrier between the shelters so crabs could only access one shelter in each trial. They were placed alternately on the side with a shelter that resulted in repeated shocks (as above) and the other side that did not result in shocks [16]. Crabs had a high probability of emerging from the shock shelter and, again, this increased over trials so that 72% emerged on the last of five trials with that shelter, but no crab emerged from a non-shock shelter. The probability of entering a shelter declined over trials but that was not affected by the nature of the shelter. That is, the crabs did not appear to discriminate between shock and non-shock shelters. However, a control group that did not receive shocks in either shelter did not reduce shelter entry. The latency to enter the shelter was greater in trials that followed shock compared with those following non-shock. Further, in this study, the latency to get out of the shock shelter was longer in the first trial compared with the final trial [16]. It seems that animals cannot discriminate between shelters with sequential presentation but can with simultaneous presentation [15]. However it demonstrates that shocks induce crabs to emerge from a dark shelter and be exposed to bright light.

These studies on *C. maenas* show that shocks markedly alter behavioural priorities. Crabs may get out of shelters following shock and in subsequent trials are less likely to enter shelters in which they had received shock (except for [16]). These changes are more pronounced with high voltages and with repeated shocks. Crabs also are hesitant about going into shelters following shock, seek alternative modes of gaining shelter by pushing at the sides of the tank, and get out of shock shelters more quickly in later trials. That is, they take risks to escape or avoid shock. They also trade-off the need to avoid bright light with the need to avoid shocks when deciding to move into a shelter or avoid it [14].

The marbled crayfish, *Procambarus virginalis*, shows negative phototaxis to white light but a positive phototaxis to blue light or darkness. Okada *et al.* [17] placed these crayfish in a T maze so that they could choose either the white light in one arm or the blue light in the other. The blue light arm was preferred to the white light arm. Then animals were trained with the blue arm fitted with metal plates so that shocks could be given when the animal entered (20 V with 3 ms duration at 50 Hz). Three training sessions were given and a later memory test, without shock, given to determine if the initial preference had changed. When shocked, the animals showed tail flick escape responses indicating the aversive nature of the shock. The majority of subjects then retreated to the normally avoided white arm and chose that arm in the memory test even if conducted 24 h after training. These results are similar to those found with shore crabs in that the shocked animals left the preferred condition and went to conditions that are normally avoided, even if tested 24 h after being shocked.

## 3. Shocks within mobile shelters

Hermit crabs, *Pagurus bernhardus*, also use shelters but these are in the form of gastropod shells and the crab can move these through the environment to forage and search for mates [18]. Hermit crabs are morphologically modified to fit the spiral shape of shells [19]. They do not abandon a shell without another being available, unless in extreme circumstances e.g. if the shell is made immobile or buried in sand or if the crab has been defeated in a shell fight [18]. Several experiments have examined the responses of these crabs to shocks delivered within a shell, to determine if the shocks alter their priorities [20–23]. The first examined how the quality of the occupied shell might affect how easy it was to make the animal abandon the shell [20]. The crabs were housed in the preferred *Littorina obtusata* or less preferred *Gibbula cineraria* shells [18]. Shocks were initiated at a low voltage and then repeated at 2 s intervals but the voltage was increased with each new shock (200 Hz, 1 s). This procedure induced the majority of crabs to get out of their shells and demonstrated the highly aversive nature of the shocks. Crabs rely on shells for protection so abandoning a shell is an extremely risky action. However, crabs in the preferred species resisted until higher voltages were given (*x* = 17.7 V) compared with those in the less preferred shells (*x* = 14.99 V). This demonstrated that abandoning the shell was not a reflex response, rather it was a behavioural decision that traded-off shock avoidance with retaining a shell of high quality.

A subsequent study again used crabs in the two species of shell but employed repeated shocks of the same intensity (10 shocks at 20 s intervals 8 V, 200 Hz, 1 s) [22]. Far fewer hermit crabs got out of their shells with these weak shocks than in Appel & Elwood [20] but more did so from the less preferred species, again showing a trade-off. Those that remained in their shells were offered a new empty shell of the same species 20 s after the cessation of shocks and their behaviour was compared with that of non-shocked crabs. Shocked crabs approached the new shell with a shorter latency, used fewer cheliped probes prior to entry and were more likely to enter the new shells. A further study by Appel & Elwood [21] reported similar changes in behaviour even when shells were offered 24 h following the shocks. These findings are consistent with the idea that the shocks within the shells caused the crabs to value their existing shell less and this changed their priorities about acquiring new shells.

Another experiment again used repeated shocks within shells of hermit crabs but some crabs were in water that contained the odour of a predator whereas the others had a non-predator odour or no added odour [23]. Single shocks were delivered for 200 ms at a frequency of 180 Hz commencing at 1 V. The voltage increased by 1 V every 10 s until either the crab evacuated from the shell or 25 V was reached. There was no difference between groups in the voltage required for the crabs to evacuate but those with the predator odour were less likely (41%) to evacuate from the shell than were those with non-predator odour (80%) or no odour group (95%). This shows that hermit crabs trade-off avoiding shocks with predation risk and this would not occur if getting out of the shell was a reflex. However, that some still got out of their shell in the presence of predator odour demonstrates a massive shift in the normal priorities for remaining within a shell.

## 4. Shocks outside of the shelter

The experiments discussed above all involved electric shocks delivered to animals within their shelters but there are other studies in which animals were shocked outside of a shelter. These were then offered access to shelter and their reactions observed. For example, Fossat *et al.* [24,25] gave repeated electric shocks to crayfish, *Procambarus clarkii*, which elicited repeated tail flip escape responses, and then placed them in a cross maze with light and dark arms, the dark arms being similar to burrows. Shocked crayfish showed a much stronger preference for the dark arms than did those that were not shocked, and the preference increased with the number of preceding shocks. The preference declined with time following cessation of shocks and was almost at the non-shocked control level after an hour. Also, Perrot-Minnot *et al.* [26] exposed amphipods, *Gammarus fossarum*, to electric shocks with 3 × 2 s pulses of 50 Hz (9 V) delivered at 5 min intervals. The amphipods reacted by performing kicking escape movements immediately following electric pulses, which suggested that electric shocks are noxious. Individual amphipods were then placed in a brightly lit tank containing an opaque refuge, which was dark within, and those exposed to shock used the refuge significantly more than did non-shocked controls. Increasing the number of electric shocks delivered in a 10 min period resulted in an increase in refuge use. Recovery from the shocks was seen after 90 min.

The results of the experiments by Fossat *et al.* [24,25] and Perrot-Minnot *et al.* [26] were interpreted as the shock inducing a state of anxiety. In support of this idea were the findings that in both species anxiolytic drugs for humans reduced the effects of shock in the crustaceans. To be maintained by natural selection, however, anxiety should have positive fitness effects and that was demonstrated by increased survival of shocked compared with non-shocked amphipods when in the presence of predatory fish [26].

## 5. Conclusions

These studies show changes in behavioural priorities that are markedly influenced by the nature of the shock, with higher voltages and repetition having much greater effects than lower voltages or single shocks. This conclusion is drawn primarily from comparing studies but one study on shore crabs used different levels of shock within the experiment and confirmed the effect [14].

Shocks within a shelter decrease the willingness of crustaceans to remain in that specific shelter whereas shock outside of a shelter increases the motivation to go into a dark shelter. This demonstrates that shocks do not cause an aversion to dark and animals shocked within a shelter avoid only that specific shelter. Indeed, we see that shore crabs learn rapidly to use an alternative shelter, if they are shocked in their original choice [15]. Shocked shore crabs will also attempt to push into the sides of a light arena before giving up those attempts and re-enter a shelter that has previously resulted in shock [14]. These observations demonstrate that shocked animals switch priorities to avoid shocks and yet maintain predator avoidance, if that option is available.

A balance between different motivational demands is usually called a trade-off. When hermit crabs decide to abandon a shell in which they had received shocks they reveal trade-offs between different priorities. They trade-off shock avoidance with retaining preferred types of shell [20,22] and also with predator avoidance [23]. However, when shore crabs abandon a shelter no trade-off between shock avoidance and light avoidance was evident, but there was a very small sample for inference about trade-offs [14]. By contrast, the hermit crab studies used repeated shocks, and that resulted in far greater numbers leaving their shells and so the sample was sufficient for meaningful analyses that showed trade-offs when leaving those shells.

However, when shore crabs made a decision about entering a shelter in which some had been shocked previously, or remaining in the light arena, the number of entries in the last five trials varied between treatments. This showed a trade-off between light avoidance and shock avoidance because both light and shock intensity affected the decision [14]. Note that decisions about entering a shelter in which shocks may have been experienced previously and decisions about leaving a shelter in which shocks are currently being experienced are different. When shore crabs entered the shelter they traded-off light avoidance with shock avoidance by using a memory of prior shock, and this cannot be a reflex. By contrast, when hermit crabs traded-off shell quality or predator cues with shock avoidance when they decided to leave their shell they were responding to the immediate problem of receiving shocks rather than memory of shocks in previous trials. While the response may superficially look like a reflex, the trade-off demonstrates that it is not. Thus, both types of trade-off are consistent with the idea of pain but do not prove pain [12,27,28]. Behavioural trade-offs, such as those described in these studies, demonstrate flexible decision-making in which animals try to make the most advantageous balance between different needs. Flexible decision-making to make the best of a poor situation requires some predictive abilities and this has been linked to the evolution of pain [27]. Investigations that examine trade-offs between shock avoidance within shelters while attempting to satisfy other requirements may provide insights to pain experience in other taxa. Trade-offs between obtaining quality food and avoidance of heat have been examined in bees [29], but using species that are normally reluctant to emerge from shelters might offer advantages in future experiments.

These studies also show that shocks have long-term effects on the behaviour of animals. First, shore crabs and crayfish learnt to use a previously avoided location to escape the shocks [15,17]. Second, hermit crabs changed their priorities about investigating alternative shells and moving into those alternative shells [22], and those changes were shown even 24 h after the noxious stimulus [21]. These shifts in priorities are not predicted by reflex reactions, rather, they are consistent with the idea of pain. Other responses following shock are also consistent with pain predictions. For example, hermit crabs that emerge from their shells show rubbing and grooming at the location of the shock on the abdomen [21]. Shore crabs and crayfish show physiological stress responses following electric shocks [24,25,30], and both species show long-term changes that have been interpreted as anxiety [14]. Further, shocks may induce shore crabs [13–16] and hermit crabs [20] to autotomize one or more walking legs, which might be mediated by a negative emotional experience [31].

Because of the long-term effects of shocks we may assume they affect the nervous system of these animals, but this has not been shown in crustaceans. Nociceptors that respond to mechanical and chemical stimuli have been noted and such stimuli elicit nerve firing in different parts of the brain in shore crabs *C. maenas* [32]. In another mandibulate arthropod, *Drosophila*, however, effects of electric shocks on nociceptive inputs have been described [33]. Shocks and thermal noxious stimuli elicit firing of separate neural pathways but then dopamine neurons integrate different noxious signals into a general aversive reinforcement pathway [32]. Such investigations would be important for understanding how different noxious stimuli influence the behaviour of crustaceans.

To conclude, the key finding of this review is that electric shocks reduce the normal high priority given to predator avoidance if that gets them away from the shock. It demonstrates the importance given to shock avoidance and it is impossible to ascribe these findings to reflexes. Rather, they indicate a marked shift in motivation that is induced by a highly aversive stimulus. Because this might expose them to risks of predation it demonstrates that the animals will pay to avoid the shocks. Similar approaches could be applied to other taxa to enable informed decisions about their potential for suffering and provide guidance for their welfare. However, it is important to note that there is no proof of pain from these studies. Instead, it is the accumulation of studies that support various pain criteria that add to the possibility of pain [5,9,10,12].

## Data Availability

This article has no additional data.
